# Correspondence

**Published:** 1882-05

**Authors:** 


### CORRESPONDENCE.

*Editor of The Independent Practitioner:*

By inserting the following notice in your journal, you will greatly oblige many members of our profession.

The Thirty-third Annual Meeting of the American Medical Association will be held at St. Paul, Minnesota, commencing Tuesday, June 6th, and lasting four days. A Section on Dentistry was formed in this Association at its last meeting, and medically educated dentists were recognized as specialists in medical science.

All medical men of the regular school, practising the specialty of dental surgery, are most cordially invited to procure credentials from their local medical societies, and join us at St. Paul. All railroads furnish reduced rates to all those members wishing to attend. A member desiring to read a paper before any section, should forward the paper, or its *title* and *length* (not to exceed twenty minutes in reading), to the Chairman of the Committee of Arrangements at least one month before the meeting. (By-Laws.) TRUMAN W. BROPHY,

*Secretary Section on Dentistry, American Medical Association.*

*Editor of the Independent Practitioner:*

Will you please announce in your journal that Dr. F. J. S. Gorgas has resigned the position of Dean and Professor in the Baltimore College of Dental Surgery, and accepted a similar position in the Dental Department of the Maryland University School of Medicine. Dr. J. H. Harris goes with him as Professor of Dental Surgery in the same school. The vacancies thus caused are filled by Dr. R. B. Winder, 140 Park Avenue, Baltimore, Md., as Dean, Dr. M. Whilldin Foster as Professor of Dental Pathology and Therapeutics, and J. B. Lindsay as Professor of Chemistry. Yours, etc., J. B. HODGKIN.

WASHINGTON, May 18, 1882.

